# Comparisons of *De Novo* Transcriptome Assemblers in Diploid and Polyploid Species Using Peanut (*Arachis* spp.) RNA-Seq Data

**DOI:** 10.1371/journal.pone.0115055

**Published:** 2014-12-31

**Authors:** Ratan Chopra, Gloria Burow, Andrew Farmer, Joann Mudge, Charles E. Simpson, Mark D. Burow

**Affiliations:** 1 Texas Tech University, Department of Plant and Soil Sciences, Lubbock, TX, 79409, United States of America; 2 USDA-ARS-CSRL, 3810 4^th^ Street, Lubbock, TX, 79415, United States of America; 3 National Center for Genome Resources, 2935 Rodeo Park Drive East, Santa Fe, NM, 87505, United States of America; 4 Texas A&M AgriLife Research, 1229 N. U.S. Highway 281, Stephenville, TX, 76401, United States of America; 5 Texas A&M AgriLife Research, 1102 East FM 1294, Lubbock, TX, 79403, United States of America; USDA-ARS-SRRC, United States of America

## Abstract

The narrow genetic base and limited genetic information on *Arachis* species have hindered the process of marker-assisted selection of peanut cultivars. However, recent developments in sequencing technologies have expanded opportunities to exploit genetic resources, and at lower cost. To use the genetic information for *Arachis* species available at the transcriptome level, it is important to have a good quality reference transcriptome. The available Tifrunner 454 FLEX transcriptome sequences have an assembly with 37,000 contigs and low N50 values of 500-751bp. Therefore, we generated *de novo* transcriptome assemblies, with about 38 million reads in the tetraploid cultivar OLin, and 16 million reads in each of the diploids, *A. duranensis* K38901 and *A. ipaënsis* KGBSPSc30076 using three different *de novo* assemblers, Trinity, SOAPdenovo-Trans and TransAByss. All these assemblers can use single *kmer* analysis, and the latter two also permit multiple *kmer* analysis. Assemblies generated for all three samples had N50 values ranging from 1278–1641 bp in *Arachis hypogaea* (AABB), 1401–1492 bp in *Arachis duranensis* (AA), and 1107–1342 bp in *Arachis ipaënsis* (BB). Comparison with legume ESTs and protein databases suggests that assemblies generated had more than 40% full length transcripts with good continuity. Also, on mapping the raw reads to each of the assemblies generated, Trinity had a high success rate in assembling sequences compared to both TransAByss and SOAPdenovo-Trans. *De novo* assembly of OLin had a greater number of contigs (67,098) and longer contig length (N50 = 1,641) compared to the Tifrunner TSA. Despite having shorter read length (2×50) than the Tifrunner 454FLEX TSA, *de novo* assembly of OLin proved superior in comparison. Assemblies generated to represent different genome combinations may serve as a valuable resource for the peanut research community.

## Background

Polyploidy is widespread in angiosperms and is thought to have been a predominant factor in their evolution and success [1]. Several important crops are relatively recently formed polyploids, including bread wheat, cotton, peanut and many more [1]. Cultivated peanut (*Arachis hypogaea*) is an allotetraploid species, whose ancestral genomes are most likely derived from the A-genome species *A. duranensis* and the B-genome species, *A. ipaënsis* [2], [3]. The very recent (several millennia) evolutionary origin of *A. hypogaea* has imposed a bottleneck for allelic and phenotypic diversity within the species [4]. However, wild diploid relatives are a rich source of alleles that could be used for crop improvement, and their simpler genomes can be more easily analyzed while providing insight into the structure of the allotetraploid peanut genome. Comparative studies conducted at the level of genetic linkage maps have revealed extensive duplication within *Arachis* species [5]. This complexity of the cultivated peanut genome and limited genetic information has affected the process of early selection of cultivars for breeding.

The genome of the cultivated peanut is thought to be ∼3 Gb, with 50,000–70,000 genes [6], and whole genome sequencing of peanut is underway. Currently, the available peanut transcriptome sequences in public databases are not complete, many have low N50 values, ranging from 500 to 750bp [4], [7], [8]. Because peanut has such a large number of genes, it is important to have a good representation of the transcriptome.

Recent advances in next-generation sequencing technology have provided opportunities for both genomic and transcriptomic studies in greater detail. In the field of sequencing, RNA-Seq and combining next sequencing of cDNA libraries has emerged as a powerful tool, which is cost-efficient and yields a far greater amount of information than does Sanger technology. RNA-Seq has been widely used to study both model and non-model organisms for SNP discovery and the identification of genes that are differentially expressed [9]–[12]. This technology provides integrated information both on expression and variants present at transcriptomic levels in complex polyploids, which in combination with ancestral diploid sequences can help characterize genic regions or transcripts of polyploids. The large amounts of sequence information from these technologies can be annotated to examine the role of genome-specific transcripts in development of complex polyploids. Furthermore, using such technologies and tools in tetraploid and diploid *Arachis* species will be a crucial step towards understanding the variants controlling complex traits and characterizing the transcripts in the complex tetraploid peanut.

For organisms with known reference genomes, mapping-first approaches have often been used for RNA-Seq analysis [12]. Reads were first mapped to the annotated references, and then assembly of transcripts, SNP identification, and the quantification of transcript expression levels were based on the mapping information. Alternatively, for those organisms lacking well-defined genomic references, these studies were typically performed using either references to related species [13], [14], assembled ESTs from multiple tissue of the target species [15]–[17], or *de novo* assembly of RNA-Seq data [7]. To use sequences from related species as references, there must be a well-studied, closely-related species. However, mapping reads to a related organism may result in a loss of information in regard to species-specific genes, and additionally, no complete overview of the target transcriptome can be generated. Assembling ESTs from the organism of interest to serve as a reference requires the existence of comprehensive EST information or a genome database. Lacking good quality references requires *de novo* assembly, which is crucial for downstream RNA-Seq analyses to gain an accurate overview of the transcriptome [12]. However, *de novo* assembly of the transcriptome has some unique challenges, particularly in the case of plants, which possess a large amount of paralogs, orthologs, homoeologs and isoforms. Assembling non-normalized transcriptomes is different from assembling normalized transcriptomes and genomes, because the read depth of transcripts is uneven, which in turn, reflects differences in expression levels. Many *de novo* assembly projects for non-model organisms have used Roche 454 pyrosequencing technology (read length currently about 500bp), because the length of reads generated are much longer than the short reads (<150bp currently) generated by Illumina's Hiseq or GAIIx technologies or ABI's SOLiD technology. However, short-read technologies are much more economical. Therefore, we have used Illumina's Hiseq and GAIIx sequencing technologies in our study.

In a previous study in peanut, transcriptome read alignment to the available 454 Tifrunner sequences indicated incomplete representation of allelic diversity due to low read depth of 454 sequencing data [18]. Also, the presence of merged gene iso-forms generated a large number of apparently heterozygous SNPs, many of which are thought to be the result of merging variants originating in homoeologous copies of the sub-genomes of peanut. It is important to separate out these gene copies to aid in better identification of homologous variants in peanut [18]. *De novo* assembly of the short reads with optimized parameters would be one way to separate the gene copies in polyploid transcriptomes such as peanut.

Recently, many *de novo* assembly programs have been developed specifically for RNA-Seq assembly using short sequence reads, and are also being applied successfully in many experiments. There are many tools that are available either freely or commercially, and which have been fairly successful in complex organisms [12], [19], [20]. Software such as SOAPdenovo-Trans [21], *AByss* [22], *Trans*-*AByss* [23], and Trinity [24] have had good success in resolving the complexity of transcriptomes. Trinity is reported to generate a high-quality *de novo* transcriptome, featuring low base error rates and the ability to capture multiple isoforms, which are crucial to maintaining acceptable levels of accuracy when characterizing genes [24]. *AByss* and *Trans-AByss* are reported to yield optimal overall assemblies, covering wide transcript expression levels by merging multiple individual *kmer* assemblies [22], [23]. SOAPdenovo-Trans is reported to provide higher contiguity, lower redundancy, and faster execution [21].

*Kmer* length that is, the length of the sequence frame used for assembly, and minimum coverage have been key factors affecting the output of *de novo* transcriptome assembly packages using de *Bruijn* graph algorithms. Assemblies constructed using single *kmer* values might result in the loss of unique contiguous sequences (contigs) and relevant biological information due to insufficient representation of kmer lengths for under-expressed genes. Using lower *kmer* values can generate a larger number of contigs, but some of them may be spurious due to sequencing errors and lack of overlap. Increasing the *kmer* values increases sensitivity and can be advantageous for differentiating homoeologs [12], [23], [25], [26], and specificity of assembling the raw reads is higher compared to lower *kmer* values. However, longer *kmer* lengths may result in fewer contigs due to capturing of only highly represented reads. A common solution to this problem is the clustering of multiple *kmer* assemblies [12].

Assembly tools have been designed for diploids including human datasets, yet many angiosperm species are polyploids. Although a few studies of *de novo* assembly have been made in polyploids [12], [14], [15], fewer have optimized parameters for polyploids. In as much as diploids and tetraploids have been used for peanut improvement, use of peanut gives an opportunity to compare *de novo* assembly and software both in diploids and tetraploids, and compare fixed to a multiple kmer analysis. In this study, we compared results obtained using three *de novo* assemblers: Trinity, SOAPdenovotrans, and TransAByss in diploid and tetraploid peanut. We also performed a multiple *kmer* analysis, which was focused on examining parameters of transcript assembly. Individual *kmer*s and clustered assemblies from Trinity and Trans*AByss* respectively, were considered for pairwise comparison to understand differences and determine a strategy that maximizes the recovery of biological information in a *de novo* transcriptome assembly of genus with different ploidy levels.

## Methods

### Plant materials

Genotypes from *Arachis* genera representing different genome combinations were selected, one tetraploid – OLin (AABB genome) [27] and two diploids – *A. ipaënsis* KGBSPSc30076 (BB), and *A. duranensis* K38901 (AA). Plants for the above mentioned genotypes were grown in the greenhouse at Texas A&M AgriLife Research under controlled conditions. Leaf, root and pod (yellow, brown and black stages of maturity) tissues were collected separately for 10 plants of each of these accessions and stored in -80°C until further use.

### RNA isolation, library construction and Illumina sequencing

Total RNA was extracted using the TRIzol reagent (Life Technologies, Grand Island, NY), followed by purification using the RNAeasy mini clean up kit (Qiagen, Valencia, CA). Tissue samples were extracted individually, RNA from leaf, pod and root was then pooled in equimolar amounts and submitted for sequencing. The quality and quantity of RNA were examined using an Agilent 2100 Bioanalyzer (Agilent Technologies, Santa Clara, CA). Complementary DNA libraries were prepared and bar-coded for each of these accessions at the National Center for Genome Resources. RNA sequencing was performed on a GAIIx Analyzer (Illumina, San Diego, CA) for the tetraploid, and on a HiSeq 2000 (Illumina, San Diego, CA) for diploids.

### Pre-assembly of short reads

*De novo* assembly was performed using SOAPdenovo-Trans, Trinity, and *Trans-AByss* at the Texas Tech High Performance Computing Center. SOAPdenovo-Trans release 1.02 03-29-2013 was used to build a *de novo* assembly with *kmer* of 25, using a minimum insert size of 200bp [21]. Trinity release 20130216 was employed with the default *kmer* of 25, minimum coverage of 2 [24]. Individual *kmer* assemblies were carried out by *AByss* version 1.3.2 with minimum mean *kmer* coverage of a unitig of 2 [22]. A minimum match percentage of 95% was selected in order to attempt to distinguish homoeologs in all three software packages. A total of ten different *kmer* assemblies with the value of 21, 23, 25, 27, 29, 31, 35, 39, 43, and 47 were built using *AByss*.

### Merging and removal of redundancy

TransAByss version 1.4.4 was used at stage 0 to merge the individual *kmer* assemblies to generate a meta-assembly with default parameters [23]. A merged multiple *kmer* assembly from *Trans-AByss* was subjected to removal of redundant sequences from the meta-assembly using CAP3 [28]. The Trinity assembly from a single *kmer* of 25 was also subjected to removal of redundant sequences to compare with the TransAByss meta-assembly. The contigs and singlets generated from the CAP3 assembler were collapsed together to form a single assembly file.

### BLASTN, BLASTX and re-mapping

Fabaceae (*Glycine, Lotus, Medicago, Phaseolus, Vigna, Cicer*, and *Arachis*) nucleotide and protein sequences were downloaded from NCBI [29], [30]. EST and protein databases were generated from the above-mentioned sequences using the *format database* command from NCBI version 2.2.28, which selected either nucleotide or inferred amino acid sequences from the GenBank entries for the seven legume genera. BLASTN and BLASTX searches for assemblies were performed versus the custom nucleotide and protein databases, respectively. BLASTN [31], [32] searches were performed with the threshold e-value of 1×10^−10^ and BLASTX [31], [32] searches were performed with the threshold e-value of 1×6^−10^ on a Supermicro 16-Opteron core server running Centos 6. Blast searches were performed on Trinity assemblies at 25 mer, Trinity at 25mer without redundant sequences and *Trans-AByss* with multiple *kmer* without redundant sequences.

After BLAST searches, assemblies were selected for remapping of the raw reads using BWA aligner [33]. Samtools were used for generating bam files and calculating statistics on the aligned files [34].

## Results

### Illumina Sequencing

To obtain an overview and for initial comparison of diploid and tetraploid peanut transcriptomes, three different genotypes, OLin (AABB), K38901 (AA), and KGBSPSc30076 (BB) were selected for paired end (PE) 2×50 bp sequencing. After filtering the raw reads, a total of 71 million 50 bp paired end reads were obtained, amounting to 34 GB of raw data for the three cDNA libraries (Table 1). Reads with an average quality of 37 were obtained, with GC percentage ranging from 43–47%, suggesting good coverage across the peanut transcriptome.

**Table 1:** Statistics on filtered FASTQ files using Fastx toolkit.

Genotype	Raw reads	Average quality	%GC content
**OLin**	38,335,246	38.00	44.44
**38901**	16,206,929	37.27	45.58
**30076**	16,774,125	37.10	47.70

### *De novo* assembly strategies

As the cultivated peanut has two sub-genomes, several assembly strategies were used and their performances in assembling the peanut transcriptome were compared. We used paired-end reads to assemble the peanut transcriptome to reduce the chance of misassembly of the large expected number of paralogs and homoeologs. We chose three state-of-the-art de *Bruijn* graph assemblers, SOAPdenovo-Trans and *Trans-AByss*, which can use multiple *kmers*, and Trinity which uses a single *kmer* to generate assemblies, respectively.

Trinity has a default *kmer* of 25 which can be changed; however, Trinity would not finish executing properly if a *kmer* value other than 25 were used. Therefore we decided to use a *kmer* length of 25 across all the three assemblers and compare the influence of assemblers at this single *kmer* value. Nine assemblies were generated using three assemblers across the three accessions and were designated as OLin_Trinity_25mer, OLin_*AByss*_25mer, OLin_SOAP_25mer, 38901_Trinity_25mer, 38901_*AByss*_25mer, 38901_SOAP_25mer, 30076_Trinity_25mer, 30076_*AByss*_25mer, 30076_SOAP_25mer (Table 2).

**Table 2:** Statistics on *de novo* assemblies generated at kmer = 25 using Trinity, AByss and SOAPdenovo-Trans.

Genotype	No. of contigs	N50 (bp)	Average (bp)
**30076_Trinity-25** ***kmer***	31,800	1,107	750
**30076-** ***AByss*** **-25** ***kmer***	29,780	1,065	746
**30076-SOAP-25** ***kmer***	37,725	900	640
**38901_Trinity-25** ***kmer***	37,379	1,401	927
**38901-** ***AByss*** **-25** ***kmer***	30,807	1,137	790
**38901-SOAP-25** ***kmer***	39,276	993	686
**OLin_Trinity-25** ***kmer***	67,098	1,641	1,112
**OLin-** ***AByss*** **-25** ***kmer***	46,003	869	655
**OLin-SOAP-25** ***kmer***	59,104	809	597

After comparing results of the nine 25 *kmer* assemblies from all three tools, we employed the multiple *kmer* strategy only on *AByss* due to easier workflow for merging *kmer* assemblies in Trans-*AByss*. Merged assemblies from Trans-AByss were designated as OLin_*AByss*_Mmer, 38901_*AByss*_Mmer, and 30076_*AByss*_Mmer (Table 3).

**Table 3:** Statistics on assemblies generated after merging multiple *kmer* assemblies using Trans-*AByss* and the non-redundant assemblies from Trinity and *AByss* [dedup – no redundant sequences, Mmer – multiple merged *kmer* assemblies].

Genotype	No. of contigs	N50	Average length (bp)
**30076_** ***AByss*** **_Mmer**	64,014	1,154	827
**30076_** ***AByss*** **_Mmer_dedup**	30,764	1,342	907
**30076_Trinity_25mer**	31,800	1,107	750
**30076_Trinity_dedup**	29,786	1,104	743
**38901_** ***AByss*** **_Mmer**	70,203	1,242	891
**38901_** ***AByss*** **_Mmer_dedup**	32,807	1,492	994
**38901_Trinity_25mer**	37,379	1,401	927
**38901_Trinity_dedup**	33,145	1,410	918
**OLin_** ***AByss*** **_Mmer**	244,372	1,203	880
**OLin_** ***AByss*** **_Mmer_dedup**	70,958	1,278	809
**OLin_Trinity_25mer**	67,098	1,641	1,112
**OLin_Trinity_dedup**	51,511	1,660	1,099

### Statistics on *de novo* assemblies

**Figure 1:**
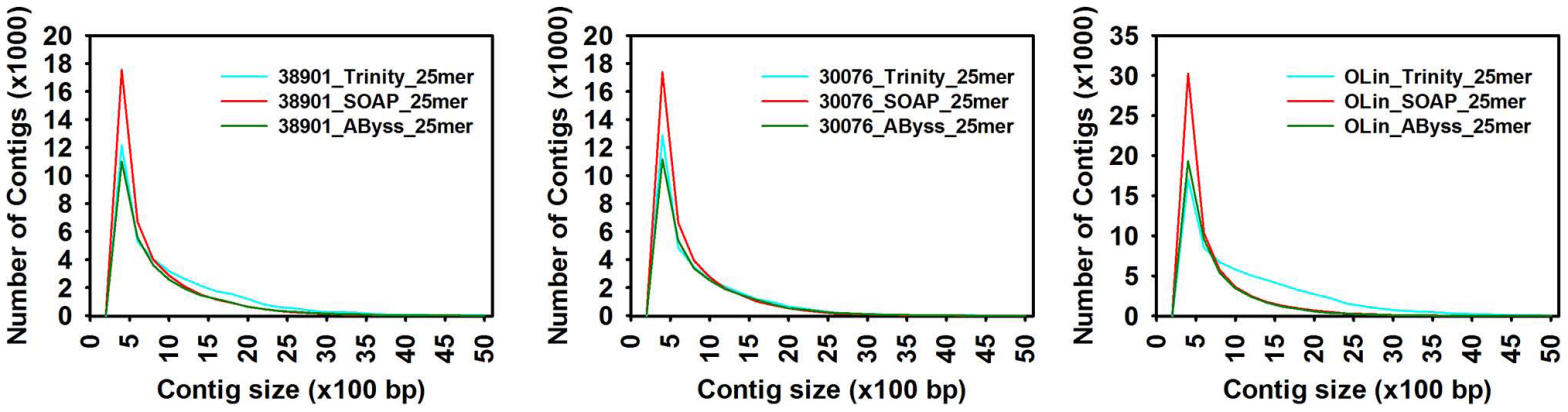
Contig length distribution generated from Trinity, AByss and SOAP at 25 kmer. Contigs greater than 200 bp were selected. A) 38901, B) 30076 and C) Olin.

**Figure 2:**
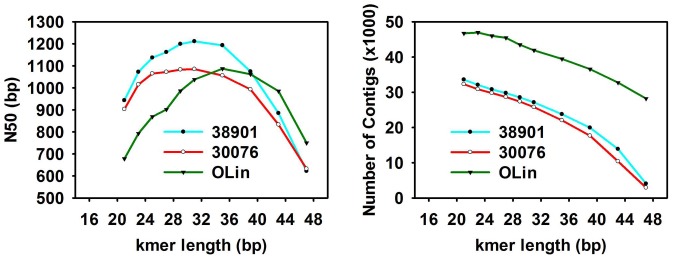
N50 and total length of the assemblies produced by AByss with different kmers. A) N50, B) contig length.

Large differences in results of kmer = 25 assemblies were identified (Table 2, Fig. 1). The N50 value from 25 kmer assemblies for OLin ranged from 809 to 1641 bp, for K38901 from 993 to 1401 bp and for KGBSPSc30076 from 900 to 1107 bp. For multiple *kmer* assemblies, N50 values increased as the read length was increased up to 35 bp for the tetraploid OLin and up to 31 bp for the diploids, and declined thereafter (Fig. 2). The number of contigs dropped as read length increased; the number of contigs present at the 35 bp kmer was 39,465 for OLin, 27,116 for K38901 and 25,772 for KGBSPSc30076 at kmer = 31 bp in the diploids.

### Merging and removal of redundancy

Merged assemblies had higher N50 values and higher numbers of contigs compared to single *kmer* assemblies of *AByss* (Table 3). On comparing the number of contigs in each of the merged assemblies, we found that each assembly had from 2.1 to 3.4 times the number of contigs in the AByss_Mmer_deduplicated assembly, suggesting the presence of additional or perhaps redundant sequences (Table 3). CAP3 [28] reduced the number of contigs by >50% in the diploids to >70% in the tetraploid. N50 values increased significantly in the diploids, and the number of contigs was reduced in all the accessions after the deduplication process. Comparing single kmer assemblies of AByss, after the removal of redundant sequences of the merged assembly, the number of contigs increased by >40% in the tetraploid compared to the kmer = 25 value (Table 2); however, there was little difference (1% to 3%) in the diploids. N50 values of the Trinity de-duplicated assembly were slightly higher in diploids than were the TransAByss de-duplicated assemblies, but were lower in tetraploid (Table 3).

### Assessment of novelty

Based on the statistics of different assemblies, we chose Trinity at 25 *kmer* (Trinity_25mer), Trinity at 25 *kmer* without redundant sequences (Trinity_25mer_dedup) and *Trans-AByss* with multiple *kmer* without redundant sequences (*AByss*_Mmer_dedup) for further assessment. After selecting a total of nine assemblies (3 species ×3 assemblies), we compared the respective genotype assemblies in a pair-wise fashion using the Mummer tool [35] which identifies the number of contigs covered by each assembly against the other (Table 4). It was observed that about from 69% to 78% of the sequences in *AByss* merged assemblies were matched in Trinity assemblies, indicating the presence of novel sequences which needs further assessment. Interestingly almost 99% of the sequences in the Trinity de-duplicated assemblies were present in the other assemblies, providing evidence of multiple gene forms separated by *de novo* approach (Table 4).

**Table 4:** Assemblies compared in a pair-wise fashion using Mummer, and the proportions covered from each of the assemblies are shown below.

	38901_AByss_Mmer_Dedup	38901_Trinity_25mer_Dedup	38901_Trinity_25mer
**38901_AByss_Mmer_Dedup**	100.00	73.81	74.13
**38901_Trinity_25mer_Dedup**	99.15	100.00	99.99
**38901_Trinity_25mer**	99.09	100.00	100.00

	30076_AByss_Mmer_Dedup	30076_Trinity_25mer_Dedup	30076_Trinity_25mer
**30076_AByss_Mmer_Dedup**	100.00	69.39	69.57
**30076_Trinity_25mer_Dedup**	99.11	100.00	100.00
**30076_Trinity_25mer**	98.99	100.00	100.00

	OLin_AByss_Mmer_Dedup	OLin_Trinity_25mer_Dedup	OLin_Trinity_25mer
**OLin_AByss_Mmer_Dedup**	100.00	77.85	78.33
**OLin_Trinity_25mer_Dedup**	98.12	100.00	99.34
**OLin_Trinity_25mer**	98.19	100.00	100.00

The upper triangular values for each accession represent the proportion of sequences at the left that were present in the sequences at the top of the triangle. The lower triangular values represent the proportion of sequences in the accession at the top that were present in the accession at the left.

### Accuracy, continuity and full length transcript estimation

On mapping the raw reads back to the assembly generated, the percentage of reads mapped was used to define the accuracy of the assembler, and continuity was defined as the BLASTN percentage match against the legume database. If the length of any of the 6 sequence reading frames matched more than 80% of the length of the reference sequence, the contig was considered to be a (potentially) full-length transcript.

**Figure 3:**
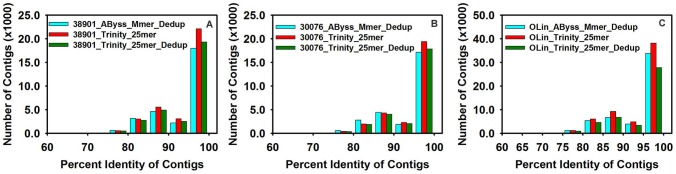
Distribution of contigs with varying percent identity against the legume EST database. BLASTN searches were performed with the threshold value of 1×10^−10^. A) 38901, B) 30076, C) Olin.

**Figure 4:**
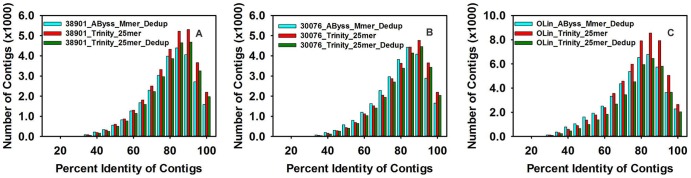
Distribution of contigs with varying percent identity against the legume protein database. BLASTX searches were performed with the threshold value of 1×10^−6^. A) 38901, B) 30076, C) Olin.

On comparing the contigs of assemblies to the NCBI legume EST database using the BLASTN program, we found from 88–92% of the contigs in each of the 6 diploid assemblies matched the BLAST database (Table 5, Fig. 3). When the tetraploid OLin was included, for Trinity match values ranged from 85–92%, but were lower (72–88%) for the *AByss* multimer. Only 72% contigs of the TransAByss de-duplicated tetraploid assembly had a match with the EST database. Comparison to the legume protein database using the BLASTX program from NCBI showed that contigs from both the Trinity assemblies had matches of >78% for OLin, >84% for K38901 and >87% for KGBSPSc30076, respectively (Fig. 4). TransAByss assemblies for diploids had 84% and 88% of contigs matched to the protein database, but the tetraploid assembly performed poorer with only 65% of the contigs being matched. Also, assemblies when compared to the legume protein database with threshold e-value of 1×10^−6^ indicated that from 43 to 47% of contigs from the Trinity assemblies and from 38 to 42% of contigs from TransAByss assemblies for diploids were full length transcripts. In case of the tetraploid, 35% of contigs from the Trinity assemblies and 25% of contigs from TransAByss were full length transcripts. Overall results from BLASTX suggested Trinity generated more full length transcripts than *AByss*.

**Table 5:** Statistics on the BLASTX, BLASTN and re-mapping of the raw reads to assemblies respectively.

Genotype	Raw reads	Reads mapped	Percent mapped	No. of contigs	Contigs with BLASTX hits	Contigs with BLASTN hits
**30076_AByss_Mmer_Dedup**	16,774,125	11,287,184	67.29	30,764	87.59	87.56
**30076_Trinity_25mer**	16,774,125	15,073,698	89.86	31,800	90.70	89.50
**30076_Trinity_25mer_Dedup**	16,774,125	15,073,380	89.86	29,786	87.32	88.26
**38901_AByss_Mmer_Dedup**	16,206,929	13,254,030	81.78	32,807	84.14	87.56
**38901_Trinity_25mer**	16,206,929	14,348,120	88.53	37,379	84.90	91.97
**38901_Trinity_25mer_Dedup**	16,206,929	14,348,119	88.53	33,145	84.75	90.65
**OLin_AByss_Mmer_Dedup**	38,335,246	31,592,825	82.41	70,958	65.43	72.48
**OLin_Trinity_25mer**	38,335,246	33,419,909	87.18	67,098	79.98	88.90
**OLin_Trinity_25mer_Dedup**	38,335,246	33,567,753	87.56	51,511	78.02	84.72

**Figure 5:**
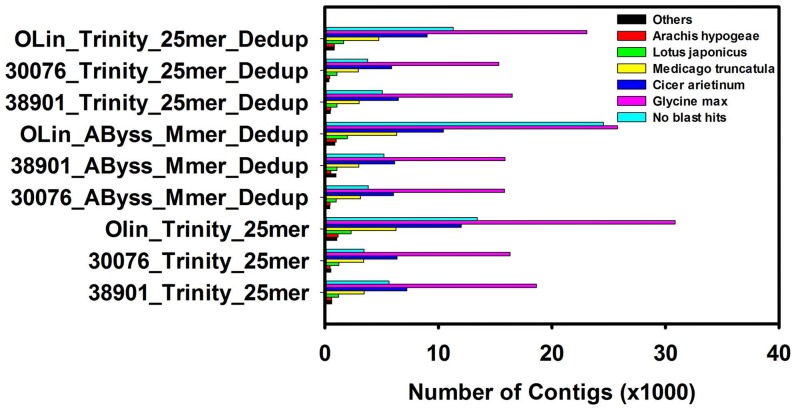
Distribution of BLASTX hits by species for the single *kmer* and non-redundant assemblies of Trinity and TransAByss.

These assemblies were assessed further to estimate the accuracy of the assembler by aligning the raw reads back to each of the assemblies. Trinity assemblies of the diploids and tetraploids had more than 87% of the reads mapping to the reference contigs, and the *AByss* multiple *kmer* de-duplicated assemblies had approximately 82% of the reads of K38901 and OLin mapping back to the reference (Table 4). BLAST results indicated each of the assemblies had the highest number of hits with *Glycine max* (Fig. 5). Although one might expect a greater number of matches to peanut, the number of peanut protein sequences in GenBank is 1,343, compared with 81,270 in soybean.

## Discussion

In this study, we generated a total of 18 assemblies, six from each genotype using different assemblers and strategies. These assemblies had promising contig lengths and N50 values (Table 2 & 3), accuracy (Table 4) and continuity (Figs. 3 & 4).

### Assembly at 25 mer using *AByss*, SOAPdenovo-Trans and Trinity

Three *de Bruijn* graph-based assemblers, Trinity, SOAPdenovo-Trans and TransAByss performed efficiently with 25 *kmer* lengths in different aspects. Trinity had better N50 values and average contig length. *AByss* had an easier approach of generating multiple *kmer* assemblies. On comparing the contig length distributions (Table 2, Fig. 2) of each of the assemblies, we found that SOAPdenovo-Trans assemblies in all the three accessions had a higher number of shorter contigs, resulting in poorer representation of the transcriptome due to lack of continuity. Trinity and *AByss* assemblies performed equally better in terms of longer contigs, but *AByss* had lesser numbers of contigs.

### Merging, redundancy removal and novelty assessment

For single *kmer* Trinity assemblies, removal of redundant sequences only slightly changed the N50 and average contig lengths in the diploids (Table 2 & 3), but the number of contigs decreased especially in the tetraploid OLin, which could reflect reporting isoforms and different splice variants [24]. Comparison of the Trinity assembly and Trinity de-duplicated assembly contigs using Mummer revealed no significant differences. But the number of sequences reduced in de-duplicated assemblies suggested the collapse of similar sequences representing different types of gene iso-forms and homoeologs.

The multiple *kmer* assembly strategy was employed only in TransAByss, and a total of 10 assemblies were generated using different *kmer* lengths ranging from 21 to 47. Contig numbers in each accession dropped as the read length was increased, and dropped more than 80% at *kmer* = 47 compared to *kmer* = 21 assemblies (Fig. 1). This could be suggesting that low-expression genes were assembled more effectively with small *kmer* sizes, leading to the assembly of numerous and highly fragmented transcripts, whereas high-expression genes were assembled more effectively with large *kmer* sizes, emphasizing contiguity. There was a tradeoff between specificity and sensitivity based on the choice of *kmer* size [12], [23], [26]. After the initial assessment of the software, we decided to use assemblies generated from *AByss* and Trinity to obtain an assembly with optimal resolution.

For the multiple *kmer* method, on merging the assemblies from *AByss* using *Trans-AByss* stage 0, we found that each merged assembly had almost 2.1 to 3.4 times more contigs (Table 3) compared to any single *kmer* assembly. The number of transcripts was higher because of the merging algorithm of *Trans-AByss,* which treats contigs as unique if they do not have nearly perfect matches [23]. On removing the redundant sequences from all the three assemblies, N50 values were approximately 15–20% higher in case of diploids but only slightly higher in the tetraploid (Table 3).

Comparing merged TransAByss assemblies to Trinity and Trinity de-deduplicated assemblies, we found that TransAByss assemblies had fewer contigs than Trinity did in the diploids, but TransAByss assemblies had about 25% more contigs based on the sequence alignment using Mummer (Table 4). This high number of contigs in the TransAByss assembly could be due to the novel transcripts reported by the process or errors generated while merging of sequences. Comparing the number of contigs in each assembly of the tetraploid to the diploid, there were approximately 1.75 to twice as many contigs in the tetraploid assemblies, reflecting the presence of two sub-genomes. This could be also suggesting that there are a significant number of homoeologs separated in the tetraploid assembly based on the number of contigs; further assessment would be required to assign tetraploid contigs to genome origin.

### Accuracy and full length transcript analysis

We used legume EST and protein databases because peanut is a legume crop, and because earlier studies in *Arachis* have shown evidence of macro synteny with *Glycine*, *Medicago* and *Lotus japonicus* [4]. We observed high similarity to these species in BLAST searches (Fig. 5). On analyzing the BLASTX and BLASTN results from each of the 9 assemblies selected above (Table 4, Figs. 3 & 4), we found that Trinity assemblies had a better continuity based on EST hits and full length transcripts based on BLASTX hits. These full length transcripts were further supported by the evidence of re-mapping of the raw reads, that these large contigs are not artifacts. Overall, the accuracy of all the assemblers was good based on the re-alignment, except for the 30076_*AByss*_Mmer_Dedup assembly (Table 3), which could be due to collapse in sequences.

Interestingly, only 67.28% of reads of the *AByss* merged assembly of 30076 mapped back to the assembly, indicating possible mis-assembly while merging the multiple *kmer* assemblies. Data obtained from BLASTX, BLASTN and remapping suggested that Trinity performed better than TransAByss in terms number of reads mapped and the percentage of contigs with BLASTN hits. In the tetraploid, TransAByss identified more contigs which could be real or artifacts, and no further analysis has been done. However, TransAByss performed poorly in the remapping of KGBSPSc30076 and in BLASTN hits for tetraploid OLin, enough to offset any advantages in the number of contigs that were present otherwise.

In the case of the tetraploid OLin_AByss_Mmer_Dedup assembly, we observed that there were approximately 24,000 contigs with no BLASTX hits, and only 65% of the reads mapped. This could be indicating the negative effect of merging multiple kmer assemblies in tetraploids. Merging the isoforms can also lead to collapse of the homoeologous sequences which would make it harder to select homologous SNPs distinguishing accessions. OLin_Trinity_25mer, 38901_Trinity_25mer and 30076_Trinity_25mer assemblies will be used as a reference for any future downstream analysis, because it would be important to have information on the isoforms and possible splice variants reported by Trinity, for differentiating the sub-genome complexities. These assemblies have been deposited at NCBI as Bioproject PRJNA248910.

## Conclusions

Given the lack of well annotated genomic resources in *Arachis* species, mapping the reads to lower quality assemblies in tetraploid species can lead to bigger challenges in downstream processing. Therefore, different short-read *de novo* assemblers were employed to obtain optimal assemblies. These assemblers proved to have a potential to assemble the peanut sequences with a higher accuracy and also provide a good overview of the transcriptome. These newer assemblies will be utilized for better SNP selection, expression analysis, mapping and QTL analysis in the tetraploid peanut. Also, it will be important to consider the best tool based on the complexity of the organism, as results from this study indicate Trinity and TransAByss gave similar results for diploids, and Trinity was better for more complex tetraploids. Overall, these assemblies representing different genome complexities may serve as a valuable reference for the peanut research community.
